# Evolution in Casting Techniques: A Narrative Review of Casting Methods, Techniques, and Innovation

**DOI:** 10.7759/cureus.53454

**Published:** 2024-02-02

**Authors:** Mila Scheinberg, Shrey Nihalani, Labdhi Mehta, Ashish Shah

**Affiliations:** 1 Orthopedic Surgery, University of Alabama at Birmingham, Birmingham, USA; 2 Orthopedics, University of Alabama at Birmingham, Birmingham, USA

**Keywords:** flexioh, casting technology, casting advancements, orthopedic casting, narrative review, casting innovation, casting methods, casting techniques

## Abstract

Orthopedic casting has seen a remarkable evolution from ancient practices to cutting-edge innovations. Beginning with ancient Egyptian methods employing bark, linen, and bandages, casting techniques have progressed through historical milestones, including the adoption of plaster of Paris in the 19th century and the introduction of synthetic materials like fiberglass and thermoplastics in the 20th century. Historical progressions transitioned from primitive materials to more sophisticated techniques, such as resin-soaked bandages and starch-based casts. While thermoplastics showcased benefits like adjustability and comfort, widespread adoption faced hurdles due to cost and water resistance limitations. The emergence of 3D printing introduced patient-specific casts with improved ventilation but faced challenges in accessibility, cost, and immediate immobilization. FlexiOH presents as a groundbreaking foam cast by Orthoheal, offering customizable fit, lightweight design, improved ventilation, and moisture resistance. Its potential to reduce ER visits, enhance patient comfort, and streamline application procedures positions it as a promising technology for the future. This paper discusses each casting generation's advantages and drawbacks, highlighting the potential of innovative technologies like FlexiOH to revolutionize orthopedic casting practices, promising improved outcomes, reduced costs, and enhanced efficiency.

## Introduction and background

The evolution of immobilization techniques, such as through casting, has remained a pivotal aspect of orthopedics, dating back to ancient Egyptian times and persisting as a key part of its history. Casting began in ancient Egypt with the use of sticks, palm fiber, and linen [1]. During the time of Hippocrates, advancements involved stiffening techniques employing wax and resin. Thereafter, the Romans stiffened casts with starch, while the first use of plaster was introduced by the Arabian surgeon Avicenna [1]. In the 1800s, plaster of Paris became a popular casting material in Europe [2]. However, its use was restricted to certain body areas, such as the lower extremities, due to the materials’ considerable weight. Since the onset of the 21st century, there have been many advancements in casting technology, such as with 3D-printed casts. An innovative solution that addresses certain limitations of 3D-printed casts is FlexiOH by Orthoheal. This immobilizer features a lightweight design, improved ventilation over traditional casting, and it is waterproof. FlexiOH is an example of the continuously evolving world of casting and exemplifies potential breakthroughs in the future of orthopedics. In this paper, we reviewed the literature to discuss and assess the evolution of historical and modern casting techniques.

## Review

Casting is a technique used in orthopedics to immobilize an injured or fractured limb during the healing process. Casting involves applying a rigid and molded material around the injured limb in order to immobilize the limb, stabilize fractures, provide support and protection, and help with recovery. Casting for medical treatment dates back to 3000 BC, with the discovery of splints made of bark, linen, and bandages found at Egyptian burial sites [1]. Hippocrates in 350 BC used wax and resin-soaked bandages to wrap injured limbs. In the 1700s, starch-based casts became popular [2]. The early 1800s marked the first use of plaster of Paris in casting with the emergence of the platre coule method in Europe, where plaster was poured in wood encasing injured limbs. In the following decades, starch paste and linen strips and plaster-infused cotton bandages became popular, which were widespread until the mid-20th century [3]. 

The introduction of synthetic casting materials, such as synthetic fiberglass and soft synthetic glass, heralded a new era in orthopedic care in the 1970s [4]. These materials, typically composed of polymeric compounds, provided advantages such as lighter weight, improved water resistance, enhanced patient comfort, customized fit, and enhanced radiolucency, facilitating treatment of complex fractures and simplifying subsequent follow-up visits [2,5,6]. This led to better patient satisfaction. Synthetic casting materials gradually gained recognition for their adaptability and superior performance.

Fourth and fifth generations

Traditional fiberglass splinting and casting led to skin complications due to water retention, leading to the development of waterproof liners [7]. Skin complications from water retention can include skin maceration, ulcers, infections, and overall pruritus and discomfort. Made from various different materials, waterproof liners are intended to serve the same purpose of reducing water retention in casted patients [8]. Gore-tex is a popular expanded polytetrafluroethylene material developed in the 1960s for industrial use and was implemented as a part of waterproof liners [9,10]. Gore-tex is traditionally used for sports gear and provides a waterproof barrier, while also being breathable and durable. 

Gore-tex lining is able to maintain a reduction in fracture similar to cotton lining with a nonsignificant difference in complication rates [7,8,11]. Due to increased freedom in lifestyle and reduced concerns about water exposure, the pediatric population prefers it [8,11,12]. Furthermore, a study used in pediatric distal radius fractures found that a hybrid mesh cast was associated with faster recovery of physical function compared to a fiberglass cast [13]. Further studies found lining changes happen less frequently in those with Gore-tex casts compared to standard cotton, allowing patients decreased trips to the emergency department or clinics saving time and resources [11]. 

One of the primary drawbacks to the universal use of waterproof liners such as Gore-tex is pricing. It can cost $30-$50 more for Gore-tex use in the cast [11]. As it is not the conventional cotton lining material, there can also be increased difficulty in properly applying the lining. Overall, waterproofing cast liners represented a large change in the casting world by creating significant quality-of-life improvements for patients, but it is not met without drawbacks. 

Initially, invented in 1964, thermoplastics were meant to be more moldable and form-fitting in comparison to older materials. Previous splints required high temperatures to solidify, whereas thermoplastics would require less heat and time [14]. Thermoplastics are primarily made of various combinations of polycaprolactone and polyisoprene, allowing for novel function [14]. Thermoplastics are lightweight, radiolucent, comfortable, and adjustable. 

Clinically, thermoplastic splints have been used for the treatment of carpal tunnel syndrome, mallet finger, and trapeziometacarpal arthritis [15]. In a systematic review of thermoplastic use in trapeziometacarpal arthritis, it was found that pain and function were improved in the majority of cases compared to neoprene splints [15]. Furthermore, a study by Al Khudiari et al. investigated the effectiveness of thermoplastic splints in nondisplaced distal radius fractures with acceptable results compared to traditional casting [16]. In mallet finger patients, thermoplastic splinting was non-inferior in radiologic outcomes compared to volar and dorsal splinting [17]. 

Thermoplastics still have certain issues that have led to decreased adoption of the technology. First, the price of these splints tends to be greater than traditional casting for multiple reasons [16]. Traditional casting materials are cheap and readily available. Thermoplastics are made of specific compounds and must undergo rigorous work and manufacturing which in turn increases the price [3]. Due to the intense process needed to make the custom thermoplastic splint, it requires specialized training to properly implement the cast [3,16]. Unlike other materials, thermoplastics are not completely water resistant leading to decreased adoption [3]. 

Although initially promising due to novel benefits, thermoplastics did not achieve widespread adoption due to significant drawbacks. It remains useful in certain cases, especially in short splinting scenarios, such as hand injuries.

Sixth generation: absolutely washable 

Furthermore, there have also been advancements in 3D printing to form unique casts for patients [13,18,19]. In recent years, 3D printed technologies are growing especially in the field of orthopedics [19]. The idea with 3D printed casts is to provide a one-to-one fit specific for the patient [20]. Other benefits of 3D-printed casts include lighter weight and improved ventilation. However, as 3D-printed casts are a pioneering technology and there have not been extensive clinical studies, there is no consensus on the materials that should be used. Chen et al. reported the first use of 3D-printed casting in a clinical trial for distal radius fractures [14]. The authors used polypropylene and polyamide as materials, which are medically compatible. Other studies have used a polylactic acid with calcium carbonate as the material for 3D casts [21]. Although there are not numerous clinical trials, early studies have shown promising results compared with traditional casting methods in patient satisfaction and decreased complications [18]. Moreover, there are several commercially available products that employ different methods of curing. Some involve injecting a curing resin into the device, while others utilize a blue light device for the process.

With that being said, 3D printed casting does not remain with drawbacks. The most prevalent hindrance in the utilization of 3D printing is the need for printers. Scanning technology is typically developed from CT or MRI findings depending on the software that is used [22]. The printers necessary for making the casts can be costly and need a high degree of expertise to operate the printers and use the 3D printed casts, which can be difficult to adopt especially for older physicians who may not be as technologically savvy. Rural communities may also suffer from a lack of accessibility as they may not have the resources to afford 3D printing technology. Certain orthopedic conditions require immediate immobilization. However, a drawback of 3D printing is the potential delay it may introduce in addressing acute or chronic conditions promptly. Moreover, issues related to the printer itself and its availability can also pose challenges in delivering timely care. Furthermore, in comparison to traditional casting methods, there is typically an increased production time for the creation of 3D-printed casts. However, as with all technologies, improvements are constantly being made. The feasibility of 3D-printed casts will just increase with time as the technology becomes more readily available [22]. This casting system represents the forefront of pioneering technology, which will only continue to improve as time moves forward.

FlexiOH

**Figure 1:**
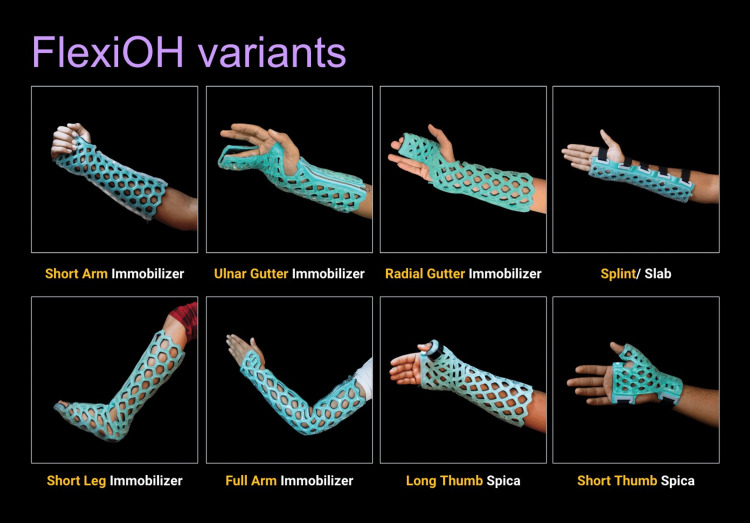
Variants of FlexiOH immobilizers Permission was obtained from the original publishers to reproduce this figure [23].

**Figure 2:**
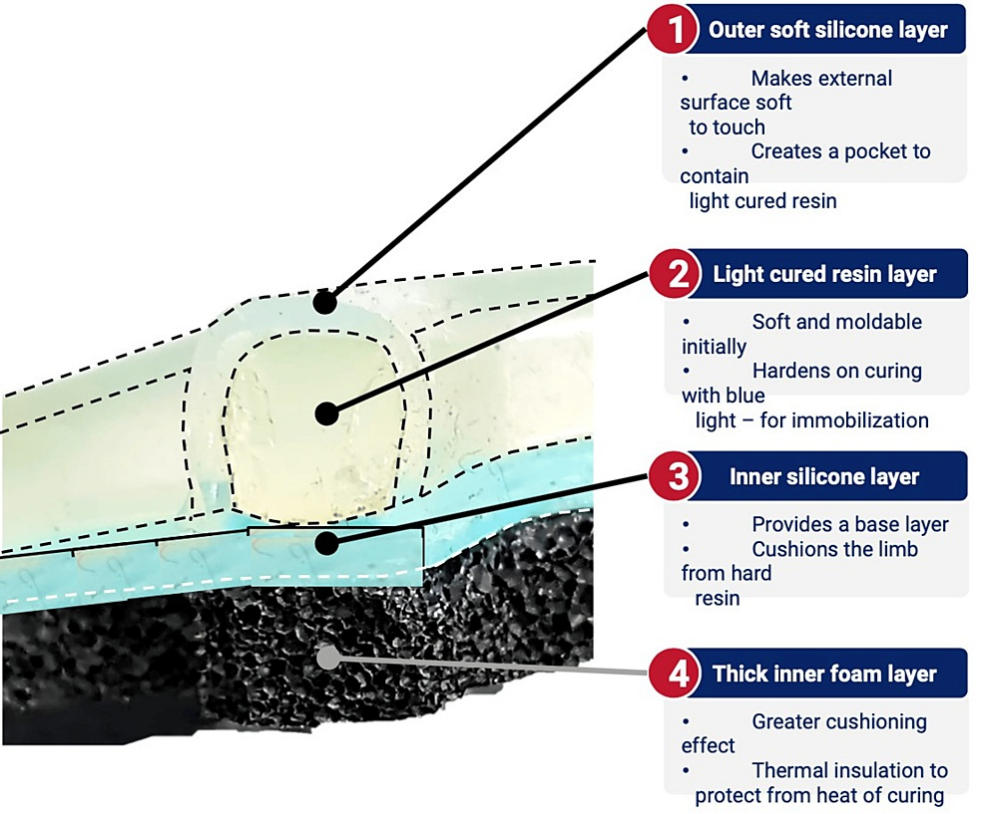
Layers of FlexiOH Permission was obtained from the original publishers to reproduce this figure [23].

**Figure 3:**
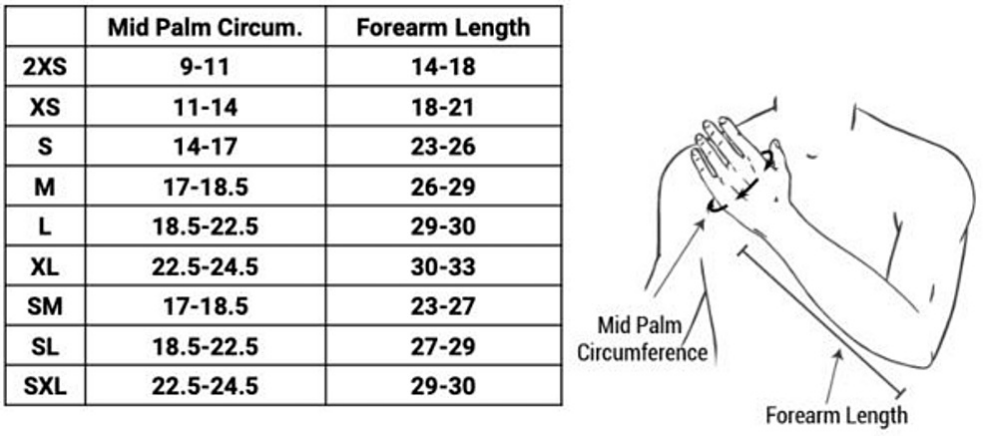
Size chart for the short-arm immobilizer Permission was obtained from the original publishers to reproduce this figure [23].

**Figure 4:**
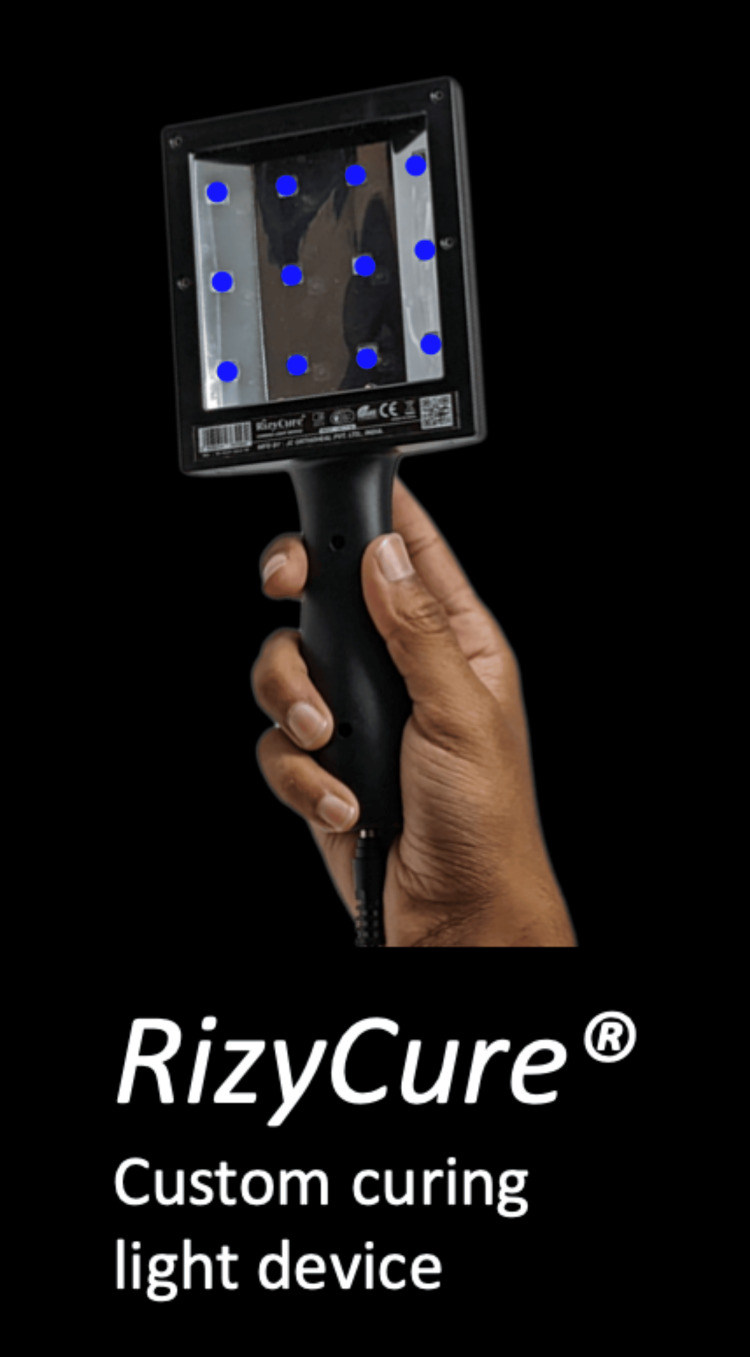
RizyCure custom curing light device Permission was obtained from the original publishers to reproduce this figure [23].

**Figure 5:**
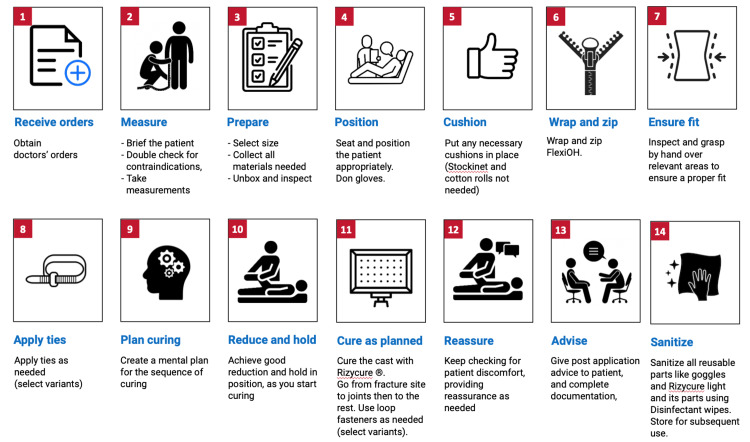
Steps of using FlexiOH Permission was obtained from the original publishers to reproduce this figure [23].

**Figure 6:**
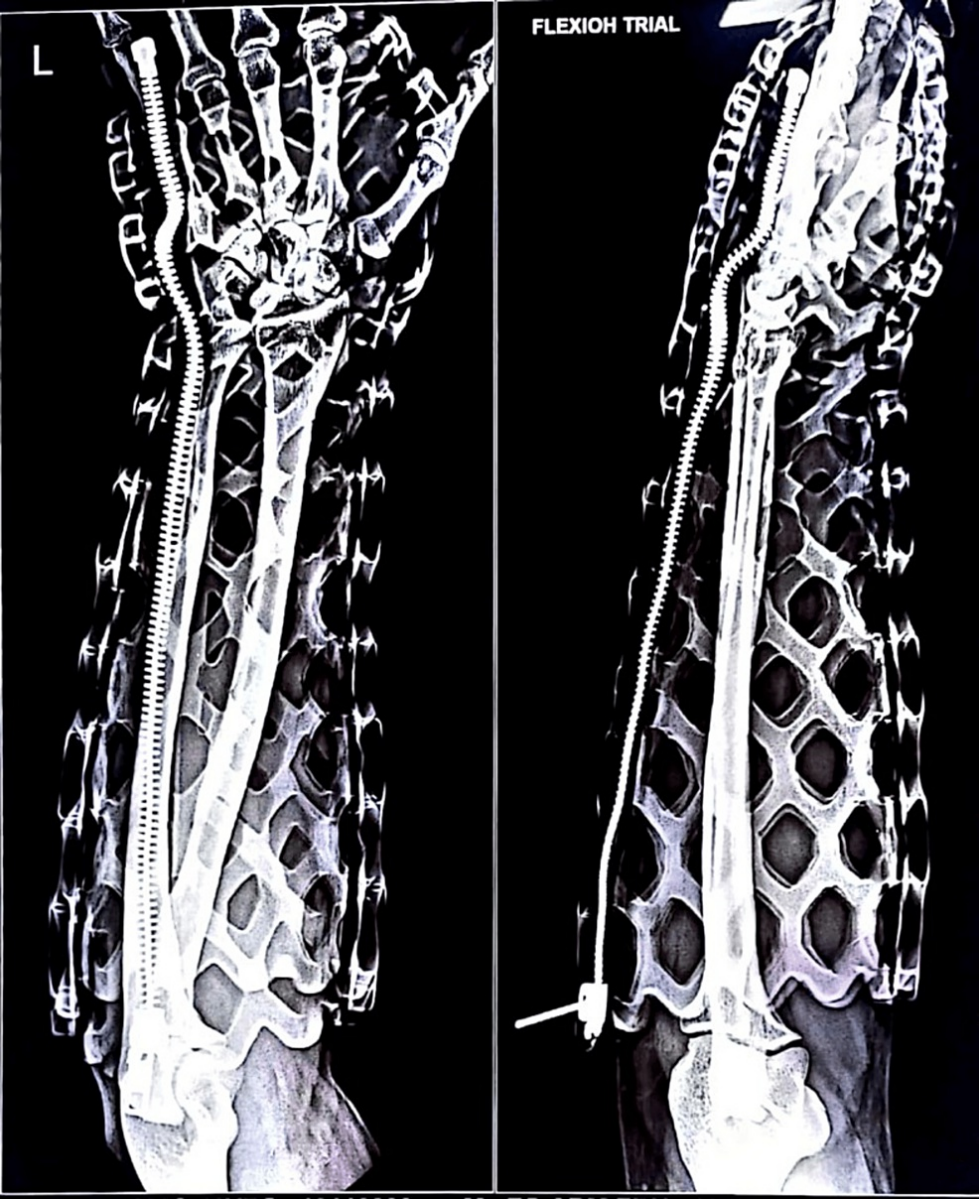
Radiograph of the short-arm FlexiOH immobilizer Permission was obtained from the original publishers to reproduce this figure [23].

New technologies are also being currently developed that seek to improve on the casting of the past. One such technology is FlexiOH by Orthoheal, which is a groundbreaking new immobilizer utilizing new polymer materials (Figure 1) [23]. FlexiOH is made of a patented foam and polymer combination allowing it to be completely customizable in fit and remain lightweight [23]. Each immobilizer consists of four basic layers: The first layer is the innermost foam layer for patient comfort, followed by a silicone layer functioning as a base layer and a cushion from the resin. The next layer on top of the inner silicone layer is the resin, which creates the structure for the immobilizer. Lastly, there is an outer silicone layer to encase the resin. Of note, the innermost layer is heat-sensitive. This means that even during the curing process, where the cast is molded using heat, the patient would not experience any discomfort or thermogenic issues. This material does not conduct heat. These layers can be visualized in Figure 2. The immobilizer must first be sized and zipped around the patient's injured limb. FlexiOH comes in multiple sizes that are based on the circumference of the limb. These measurements are compared to a size chart to aid in selecting the correct size of FlexiOH immobilizer. An example of a sizing chart for a short-arm immobilizer can be seen in Figure 3. Once the immobilizer has been adequately fit to the patient, it hardens with the use of the RizyCure™ blue light device [23] (Figure 4) while the patient's fracture is reduced. The cast can be shaped to maintain the fracture reduction while also accommodating the individual's unique anatomy, including any bony prominences and the specific shape of the limb. Furthermore, its unique design with hollow spaces allows water and sweat to evaporate more freely [23]. A step-by-step user guide can be found in Figure 5. The immobilizer is radio-opaque and can be visualized on X-ray, as can be seen in Figure 6.

FlexiOH is especially useful for pediatric populations because the zip closure for the immobilizers helps avoid the fright of cast saws. Applying FlexiOH is straightforward and can be learned through a simple training session with the device. This proves valuable in settings where trained casting personnel may be scarce, such as in the ER or remote locations. In addition, it offers a cost-effective solution. FlexiOH is a promising new technology that remains to be clinically tested. 

While the commercially available products made by FlexiOH are currently only targeting specific parts of the body, this technology has a lot of potential in areas, such as the foot and ankle, knee, spine, and shoulder. Specifically, in the foot and ankle area, FlexiOH could be helpful in patients with diabetic neuropathy and Charcot syndrome to replace regular casting and fiber casting, which can increase complications, such as ulceration in 40% of the population [24]. FlexiOH allows for visibility of the skin, which can help reduce these soft tissue complications. With the unique FlexiOH technology, this splint gives better patient comfort, breathability, and washability and is easier to place. FlexiOH is also removable during rehabilitation.

Another flaw with traditional casting and total contact casting is the high number of emergency room (ER) visits secondary to wet or loose casts. A study done by Sawyer et al. showed that “of 168 ER visits made by 155 children treated with cast immobilization, 29% were because of a wet cast; 10%, a damaged cast; 23%, a tight cast; 13%, a loose cast; and 10%, pain. In addition to wet and damaged casts, compliance issues included a missed clinic appointment (5%) and being told by medical personnel to return to the ER for a cast check (8%)” [25]. The innovative FlexiOH technology offers a promising solution to mitigate these ER visits. It effectively addresses the challenge of wet casts by virtue of its waterproof design. In addition, when it comes to visits prompted by discomfort or a tight cast, FlexiOH provides an adaptable solution. Unlike the conventional approach of hospital-based bivalving for pain relief, adjustments, or the dreaded concern for compartment syndrome, FlexiOH offers a user-friendly alternative: it can be effortlessly unzipped to achieve a more comfortable fit. A tight case can be bivalved by unzipping the cast, eliminating a visit to the ER for a bivalve, which can help save healthcare dollars and time. This technology can not only reduce readmission rates and emergency room visits, but it also helps patients avoid ulceration and long-standing side effects from casting. 

In addition to decreasing the number of ER visits, this innovative technology also proves highly beneficial in terms of time efficiency for both physicians and patients, notably by minimizing ER wait times. In acute trauma situations involving upper and lower limb injuries, ER physicians are not required to be as hands-on as they would be with traditional casting, which can take up to 35-40 minutes to apply. This affords them the opportunity to attend to other patients during that time.

The conventional method of applying casts is both time-consuming and relies heavily on the skill of the operator. Obtaining a specialized cast technician, particularly in rural or smaller healthcare settings, can be challenging due to factors, such as cost and resource availability [26]. By contrast, the introduction of FlexiOH technology significantly reduces the learning curve, minimizing potential issues related to suboptimal casting techniques. This makes it an invaluable asset in such settings. With the rising costs of casting materials and specialized personnel, this technology proves cost-effective as it not only saves time but also reduces the increasing labor expenses. Moreover, it enhances resource availability, which can be highly beneficial in this context.

This technology holds the potential for revolutionizing acute trauma management in high-stress environments like warzones and mass casualty scenarios. It allows for swift immobilization, as it can be applied by individuals without specialized cast training, taking only three to six minutes per limb. This proves invaluable in situations where time is critical and resources may be limited. In addition, it could significantly enhance wound care management.

Certain types of casting, such as hip spica, as well as advancements in other areas like spine, knee, and shoulder immobilization in adults, can be laborious, time-sensitive, and require specialized personnel. Factors like personal hygiene also come into play. As the FlexiOH technology is waterproof and open, this will help with hygiene issues. Therefore, any significant progress in using this technology for these sorts of cases could potentially bring about a revolutionary change in the field.

The advancement in technology, particularly in products like FlexiOH, holds the potential to significantly improve cost-effectiveness, ensure consistent and reliable outcomes, and minimize errors in application. FlexiOH is FDA listed [23]. This innovation has the capacity to streamline the casting process, making it more accessible and efficient across various healthcare settings. FlexiOH of course has some limitations, including constraints related to access, the requirement for certain fracture types, and the necessity for fit within a predefined set of options, limiting its full customization. Another main limitation is the current lack of biomechanical research for this product, which warrants future studies.

## Conclusions

The process of casting has advanced significantly since the advent of plaster of Paris. With that being said, it remains a mainstay for many providers due to ease of use and lower costs in comparison to more modern methods. Similar to the advancements seen in orthopedic implants and surgical technology, innovative products like FlexiOH have the potential to revolutionize the field. They offer advantages such as simplified application, fewer complications related to casting, potential reductions in readmissions and ER visits, and long-term cost savings for healthcare. This technology holds great promise for the bracing industry. The new frontier of casting and immobilization is exciting as it has the ability to change our current paradigm.
